# Complete genome sequence and anti-obesity potential of *Lactiplantibacillus plantarum* HOM2217 in 3T3-L1 cells and high-fat diet-fed rats

**DOI:** 10.3389/fmicb.2024.1436378

**Published:** 2024-09-11

**Authors:** Tingting Wang, Xiao Zhang, Linlin Fan, Ying Zhao, Zhengwen Zhang, Zhonghua Cao, Ying Xu, Suwon Lee, Chongyoon Lim, Shiqi Zhang

**Affiliations:** ^1^Research Center, Beijing Hanmi Pharmaceutical Co., Ltd., Beijing, China; ^2^Food & Biotech R&D Center, Coree Beijing Co., Ltd., Beijing, China

**Keywords:** *Lactiplantibacillus plantarum*, complete genome sequence, 3T3-L1 cells, anti-obesity effect, high-fat diet

## Abstract

The global prevalence of obesity is rising year by year, which has become a public health problem worldwide. Many animal and clinical studies have shown that *Lactiplantibacillus plantarum* is considered an ideal probiotic and potential supplement for the treatment of obesity. In this study, we aimed to complete the genome sequence of *L. plantarum* HOM2217, which was isolated from human milk, and study its physiological characteristics and anti-obesity effects in 3T3-L1 cells and rats fed a high-fat diet (HFD) to determine its potential as a starter for functional food products. Whole-genome analysis demonstrated that HOM2217 contained a single circular chromosome of 3,267,529 bp with a GC content of 44.5% and one plasmid (62,350 bp) with a GC content of 38.5%. Compared to the reference strains, HOM2217 demonstrated superior tolerance to gastrointestinal conditions, higher adhesion to intestinal epithelial cell lines, potent antimicrobial activity against *Enterobacter cloacae* ATCC 13047, and effective cholesterol removal ability *in vitro*. Treatment with heat-killed HOM2217 significantly reduced lipid accumulation and intracellular triglyceride production in 3T3-L1 adipocytes. Daily treatment of HFD-fed rats with HOM2217 for 7 weeks decreased body weight, body weight gain, and body fat without changes in food intake. HOM2217 also significantly increased the serum high-density lipoprotein cholesterol (HDL-C) level, decreased the serum tumor necrosis factor (TNF-α) and increased short-chain fatty acid (SCFA) (formic acid, acetic acid, and butyric acid) levels in the cecum. Thus, HOM2217 could potentially prevent obesity in rats by inhibiting inflammatory responses and regulating lipid metabolism and SCFAs expression. Therefore, HOM2217 has potential as an alternative treatment for obesity.

## 1 Introduction

According to the World Health Organization, overweight and obesity are defined as abnormal or excessive fat accumulation that poses health risks. A body mass index (BMI) > 25 is considered overweight, and > 30 obese (WHO, 1997). In 2022, 2.5 billion adults aged 18 years and older were overweight, including over 890 million adults who were living with obesity (Phelps et al., 2024). Obesity has now reached epidemic proportions and it is estimated that by 2030, more than one billion adults worldwide will become obese (World Obesity Federation, 2022). Obesity is classified as a low-grade chronic and systemic inflammatory disease that results from complex interactions between genes and environmental factors and leads or relates to many diseases such as type 2 diabetes (Qin et al., 2020; Yu et al., 2022), nonalcoholic fatty liver disease (Quek et al., 2023), cardiovascular disease (Dwivedi et al., 2020; Opio et al., 2020; Mohammadian Khonsari et al., 2022), chronic kidney disease (Kjaergaard et al., 2022), thyroid disorders (Song et al., 2019), COVID-19 (Hussain et al., 2020; Raeisi et al., 2022), asthma (Scott et al., 2023), obstructive sleep apnea (Ghavami et al., 2023), stroke (Wang et al., 2022), osteoarthritis (Ortolan et al., 2021), and cancer (Petrelli et al., 2021; Lee et al., 2022). Moreover, it affects the quality of life. Currently, methods to treat obesity mainly include dietary regulation, exercise, drug administration, and bariatric surgery (American College of Cardiology/American Heart Association Task Force on Practice Guidelines, Obesity Expert Panel, 2013, 2014; Apovian et al., 2015; Garvey et al., 2016; Reid and Korner, 2022). Pharmacotherapy is indicated as an adjunct to a reduced-calorie diet and increased activity for long-term weight management (Yanovski and Yanovski, 2014; Apovian et al., 2015). However, some drugs may increase the incidence of gastroparesis (Wilding et al., 2021) and pulse rate (Pi-Sunyer et al., 2015) or attenuate the expected blood pressure reduction (Pi-Sunyer et al., 2015). Although Semaglutide, the latest breakthrough in anti-obesity medication, represents a significant advancement in obesity treatment, it also carries common side effects such as nausea, vomiting, diarrhea, constipation, injection site reactions, along with rare potential risks of thyroid tumors and pancreatitis (Wilding et al., 2021).

Obesity and its association with other chronic noncommunicable diseases are not only from genetic factors, eating habits, or lack of physical activity but also from the gut microbiota as an environmental factor (Alvarez-Arrano and Martin-Pelaez, 2021). Obesity is associated with perturbation of the gut microbiome (Ley et al., 2006), which affects metabolic function and energy homeostasis (Guirro et al., 2019) by affecting energy use, nutrient metabolism, inflammatory pathways, and the gut-brain axis (Rawat et al., 2023). Therefore, novel therapies targeting the gut microbiome, such as fecal microbiota transplantation and supplementation with probiotics and prebiotics, are potential treatments for obesity (Asadi et al., 2022). The consensus definition of probiotics is: “live microorganisms that, when administered in adequate amounts, confer a health benefit on the host” (Hill et al., 2014). Probiotics exert health effects via a variety of mechanisms, including the modulation of immune function, production of organic acids and antimicrobial compounds, interaction with resident microbiota, interfacing with the host, and improving gut barrier integrity and enzyme production (Sanders et al., 2019). Currently, members of the genera *Lactobacillus* and *Bifidobacterium* are primarily used.

*Lactiplantibacillus plantarum*, previously known as *Lactobacillus plantarum*, an important member of *Lactobacillus,* is a well-studied and versatile species with beneficial effects, such as promoting host immunity (Zhao et al., 2021), reducing atopic dermatitis (Prakoeswa et al., 2017), improving functional diarrhea (Jung et al., 2022) and irritable bowel syndrome symptom severity (Martoni et al., 2023), conferring antioxidant, anti-cholesterolemic and anti-glycemic effect (Oh et al., 2021; Stsepetova et al., 2023). It has a long tradition of safe use and enduring relationship with humans. In clinical studies, *L. plantarum* has been shown to have effective probiotic anti-obesity effects (Park et al., 2020; Rahayu et al., 2021; Sohn et al., 2023). However accumulating evidence indicates that probiotic effects may vary in a strain-and dose-specific manner (McFarland et al., 2018; Ji et al., 2019).

Therefore, in this study, we isolated the specific HOM2217 strain from human milk, analyzed its complete genome sequence, and evaluated its tolerance to gastrointestinal conditions (acid and bile), adhesion to the gastrointestinal epithelium, prevention of obesity, and cholesterol-lowering ability. We investigated its potential anti-obesity activity in 3T3-L1 cells and its effects on body weight, body fat, and serum lipid and cytokine concentrations in rats for the development of new functional food products.

## 2 Materials and methods

### 2.1 Bacterial strains and culture conditions

HOM2217 was isolated from human milk and deposited at the China General Microbiological Culture Collection Center (CGMCC; No. 25683). *Enterobacter cloacae* ATCC 13047, *Lactocaseibacillus rhamnosus* ATCC 53103 (GG), and *L. plantarum* ATCC 14917 were purchased from the American Type Culture Collection (ATCC) and used as reference strains for comparison of phenotypic properties. HOM2217, *L. plantarum* ATCC 14917, and *L. rhamnosus* ATCC 53103 (GG) were grown in de Man, Rogosa, and Sharpe (MRS) broth (OXOID, USA) and *E. cloacae* ATCC 13047 was grown in Tryptone Soya Broth (OXOID, USA) at 37°C for 20 h aerobically freshly before use. Lyophilized HOM2217 powder was used in animal experiments. First, it was fermented in a 30-L fermentation tank at 37°C at constant pH 5.5 and a stirring speed of 50 rpm for 20 h. Then, the cells were harvested by centrifugation at 10,000 × *g*, 4°C for 10 min, washed twice with sterile saline solution, resuspended in cytoprotectant, lyophilized for 48 h, and stored at −20°C until use. The lyophilized HOM2217 powder was freshly suspended in sterile saline solution daily for administration to animals.

### 2.2 Whole genome and comparative analysis

The HOM2217 genome was sequenced using the Illumina II and PacBio Sequel III platforms (Majorbio, Shanghai, China). The bacterial genome completion map was assembled *de novo* using Unicycler v0.4.8 (Wick et al., 2017) and error-corrected using Pilon v1.22. Gene coding sequences (CDS), tRNA, rRNA, and sRNA were predicted using Glimmer v3.02 (Delcher et al., 2007), GeneMarkS v4.3 (Besemer and Borodovsky, 2005), tRNAscan-SE v2.0 (Chan and Lowe, 2019), Barrnap v0.9, and Infernal v1.1.4, respectively. Genome visualization was performed using Circos v0.69.6 (Stothard and Wishart, 2005). The corresponding function annotation was completed by blasting genes against the Cluster of Orthologous Groups of proteins (COG) database (Jensen et al., 2008). The complete genome of HOM2217 was submitted to GenBank (GenBank accession number: CP152360.1).

For comparative genomic analysis, the genome sequences of nine *L. plantarum* strains [Dad-13 (GCA_023547165.1), LMT1-48 (CP033888.1), UBLP-40 (GCA_003692725.1), P-8 (CP005942.1), Q180 (CP073753.1), HAC01 (CP029349.1), EM (CP037429.1), 299v (GCA_001888735.1), and ATCC 14917 (GCA_000143745.1)] were obtained from the NCBI database and used as references. An orthologous gene set was built to identify the core genome and pan-genome sizes using OrthoMCL package v2.0.9 (Li et al., 2003). A maximum-likelihood (ML) tree with bootstrap values (1,000 replications) based on the concatenated nucleotide sequences of homologous single-copy genes was constructed using MEGA7 software (Kumar et al., 2016). The average nucleotide identity of the genome sequence between HOM2217 and other reference strains was evaluated using the orthologous average nucleotide identity (Ortho ANI) tool (Zhang et al., 2019).

### 2.3 Survival under simulated gastrointestinal tract conditions

Tolerance to gastrointestinal tract (GIT) conditions was evaluated using simulated gastric and intestinal juices based on the methods described by Kang et al. (2020) and Zhang et al. (2022), with some modifications. Briefly, 1 mL of the 20-h culture was harvested by centrifugation at 10,000 × *g*, 4°C for 10 min, and the cells were washed twice with phosphate buffer solution (PBS, Thermo Fisher, USA) to obtain a cell concentration of 1 × 10^8^ CFU/mL. One milliliter of the cell suspensions was inoculated into 9 mL of simulated gastric juice composed of 10 g/L pepsin (Sigma-Aldrich, USA) with pH was adjusted to 3.0 and incubated at 37°C for 3 h. After incubation, the bacteria were centrifuged and resuspended in 10 mL of simulated small intestinal juice which was composed of 10 g/L trypsin (Sigma-Aldrich, USA), 3 g/L oxgall (Difco, USA) and 6.8 g/L KH_2_PO_4_ (Sigma-Aldrich, USA) with pH was adjusted to 6.8 and incubated at 37°C for 24 h sequentially. The GIT tolerance of cells was determined by viable cell counts on MRS agar plates. The percentage of bacterial survival was calculated using the following equation: survival rate (%) = final (log CFU/mL)/initial (log CFU/mL) × 100%.

### 2.4 Adhesion ability to Caco-2 cells

Caco-2 cells were obtained from the cell bank of Chinese Academy of Sciences and grown in Dulbecco’s Modified Eagle Medium (DMEM, Thermo Fisher, USA) supplemented with 10% (v/v) fetal bovine serum (FBS, Corning, New Zealand) and antibiotics (100 U/mL penicillin, 100 μg/mL streptomycin, Thermo Fisher, USA) at 37°C in an atmosphere of 5% CO_2_ at constant humidity. The adherence assay was performed as described by Zhang et al. (2016) and Sophatha et al. (2020), with some modifications. Briefly, cells were seeded at approximately 2 × 10^5^ cells/ml into 24-well cell culture plates (Corning, USA), and the medium was changed every 2 days until the cells were fully differentiated at 14 days. A fresh strain suspension (1 × 10^8^ CFU/mL) which was suspended in 1 mL of DMEM with 10% (v/v) fetal bovine serum (FBS, Corning, New Zealand) without antibiotics, was added to the previously prepared Caco-2 cell monolayers. The cell mixtures were incubated at 37°C under 5% CO_2_ atmosphere for 2 h, and then each well was gently washed three times with PBS solution to remove non-adhering bacteria. The Caco-2 cells and adhering bacteria were disrupted using 0.1%TritonX-100 (Nanjing Jiancheng, China) and bacterial counts were performed on MRS agar media. Adhesion capacities were expressed as the percentage of bacteria that adhered to the total number of bacteria initially added.

### 2.5 Assessment of antimicrobial activity

The antimicrobial activity of HOM2217 against *E. cloacae* ATCC 13047 was performed as described by Choi et al. (2019) with some modifications. Briefly, the strain cultures were harvested by centrifugation at 10,000 × *g*, 4°C for 10 min and their Cell-free supernatants (CFS) were filtered through 0.22 μm membrane filter. CFS at different concentrations (0, 5, 10, 15, and 20%) were cocultured in the presence of *E. cloacae* ATCC 13047 (10^6^ CFU/mL) at 37°C for 24 h. *E. cloacae* ATCC 13047 cells were used to evaluate the antimicrobial activity. The same concentrations of LGG cell-free supernatant were used as positive controls.

### 2.6 Assimilation of cholesterol

Cholesterol was evaluated using the procedure described by Albano et al. (2018) with some modifications. Briefly, freshly prepared MRS broth was supplemented with 0.30% ox gall. Water-soluble cholesterol (Cholesterol-PEG 600, Sigma, USA) was filter-sterilized and added to the broth at a final concentration of 120 μg/mL, inoculated with each strain, and incubated anaerobically at 37°C for 24 h. Following incubation, bacterial cells were harvested by centrifugation (10,000 × *g*, 10 min). The supernatant and uninoculated control MRS broth were assayed calorimetrically to determine cholesterol content using the o-phthalaldehyde method described by Shehata et al. (2016).

### 2.7 Assessment of the inhibitory effect on 3T3-L1 adipocytes

3T3-L1 cells were obtained from the cell bank of Peking Union Medical College (PUMC, China) and cell viability was evaluated using the CellTiter 96^®^ Aqueous One Solution Cell Proliferation Assay kit (Promega, USA) (Park et al., 2011). 3T3-L1 cells were cultured as described by Jeung et al. (2018) with some modifications. Briefly, 3T3-L1 cells were seeded at a density of 2 × 10^5^ cells/well in 24-well culture dishes until confluence was reached. Two days post-confluence (D0), the medium was replaced with differentiation cocktail medium (MDI: 0.5 mM 3-isobutyl-1-methylxanthine (IBMX; Sigma, USA), 1 μM dexamethasone (Dex; Sigma, USA), and 10 μg/mL insulin (Sigma, USA)) in DMEM supplemented with 10% FBS, and the cells were incubated for 4 days. On day four (D4), the medium was replaced with DMEM containing 10 μg/mL insulin (Sigma, USA) and the cells were incubated for 2 days. On day six (D6), the medium was replaced with DMEM and the cells were incubated for 2 days. Differentiation was completed on day eight (D8). Heat-killed strains (70°C, 30 min) were treated with the medium from D0 to D8. Lipid accumulation was quantified using Oil Red O (Nanjing Jiancheng, China) according to the manufacturer’s instructions. The cells were observed under an optical microscope. Oil Red O was quantified by extracting the dye with 100% isopropanol and measuring the absorbance at 500 nm using a microplate reader. TG and protein concentrations were measured using a commercial TG and TP assay kit (Nanjing Jiancheng, China) according to the manufacturer’s instructions.

### 2.8 Anti-obesity effect in diet-induced obese rats

The experimental animals comprised 70 males eight-week-old Sprague–Dawley rats purchased from Beijing HFK Bioscience Co., LTD (China). After 1 week of adaptation to solid formula feed (Beijing HFK Bioscience Co., LTD, China) at the Lab Animal Center of Beijing Union University, the animals were randomized according to their body weights and divided into two groups. Rats were fed either a high-fat diet (HFD; *n* = 60) or a normal diet (ND; *n* = 10) for 2 weeks. Energy from fat was 60 and 10% for the high-fat and normal diets, respectively. After 2 weeks of feeding, the rats fed a high-fat diet were sorted according to body weight gain, and one third of the rats that were obesity-resistant rats and exhibited lower weight gain were eliminated. Forty obesity-sensitive rats were randomly divided into four groups according to body weight: a model control group (HFD) and three treatment groups, high-fat diet with low dose HOM2217 (HFDL), high-fat diet with medium dose HOM2217 (HFDM), and high-fat diet with high dose HOM2217 (HFDH), with 10 rats in each group.

HOM2217 viable bacteria for animal administration were prepared by suspending the lyophilized bacterial powder in PBS. Clinical trials indicate that *L. plantarum* doses for humans range from 1 × 10^10^ to 2 × 10^11^ CFU/day, with a recommended low dose of 2.5 × 10^10^ CFU/day. In the study, rats in the HFDL, HFDM and HFDH groups received 2.5 × 10^9^, 1.25 × 10^10^, and 2.5 × 10^10^ CFU/kg body weight, respectively, for 7 weeks, with doses converted from human levels using a 6.0-fold adjustment factor based on a 60 kg human weight (Kadooka et al., 2010; Michael et al., 2021; Nasiri et al., 2021; Oh et al., 2023).

The ND and HFD groups received the same volume of the vehicle. The well-being, food intake, and body weight of the animals were recorded weekly. The body weight gain was calculated. At the end of the experiment, the rats were fasted overnight and euthanized by CO_2_ asphyxiation. The body fat (epididymal and perirenal fat pads) was excised and weighed. Serum was separated from the blood by centrifugation at 1,200 × *g* for 10 min at 4°C and stored at −80°C until analyses. Serum TC, TG, HDL-C, and LDL-C levels were measured using a Cobas C311 Chemistry Fully Automatic Biochemistry Analyzer (Roche, Switzerland), according to the manufacturer’s protocols. Tumor necrosis factor-α (TNF-α) and interleukin 6 (IL-6) levels were determined using the rat ELISA Kit (RayBio, USA) according to the manufacturer’s instructions. Approximately 1,000 mg of cecal contents were removed, and the levels of SCFAs were determined by gas chromatography–mass spectrometry (GC–MS) (Agilent 8860, USA) using a previously described method (Li et al., 2020).

### 2.9 Statistical analysis

All *in vitro* experiments were repeated in triplicate. Experimental values are expressed as mean ± SD or mean ± SEM. Data were analyzed using either Student’s t-test or one-way analysis of variance (ANOVA), followed by Dunnett’s *post hoc* correction for multiple comparisons in GraphPad Prism (Version 9.0, GraphPad Software Inc., San Diego, CA, USA), and statistical significance was set at *p* < 0.05.

## 3 Results

### 3.1 General genome features of HOM2217

**Figure 1:**
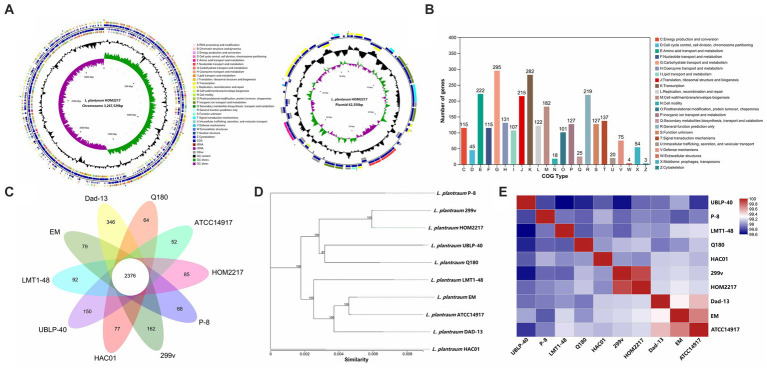
Genome features of HOM2217. **(A)** Circular genomic map of HOM2217. The circular map was generated using Circos and contains seven circles. Marked information is displayed from the outer circle to the innermost circle as follows: Genome size, CDSs on the forward stand, CDSs on the reverse stand, prophage regions, rRNA and tRNA, GC content, and GC skew. **(B)** Gene number of COG categories. **(C)** Numbers of orthologous gene families and unique genes among 10 *L. plantarum* strains. The Venn diagram shows the number of orthologous gene families of the core genome (center) and the numbers of unique genes of each genome. The different colors indicate different sampling areas of the strains as indicated. The orthologous gene families were determined using OrthoMCL software with an inflation value of 1.5. **(D)** Phylogenetic tree of 10 *L. plantarum* strains. The phylogenetic tree was built based on aligned concatenated sequences of single copy orthologous gene families. The bootstrap support value before each node represents the confidence degree of each branch. **(E)** Average nucleotide identity (ANI) tree analysis of 10 *L. plantarum* strains.

As shown in Figure 1A, the complete genome of HOM2217 contained a single circular chromosome of 3,267,529 bp with a GC content of 44.5%, and one plasmid (62,350 bp) with a GC content of 38.5%. A total of 2,741 genes were classified into COG functional categories (Figure 1B). Among these, a total of 295 genes (10.8%) were involved in carbohydrate transport and metabolism, 282 genes (10.3%) in transcription, 222 (8.1%) in amino acid transport and metabolism, 107 (3.9%) in lipid transport and metabolism, and 219 genes (8.0%) were involved in general function prediction. *L. plantarum* HOM2217 contains genes related to acid and bile tolerance, epithelial cell adhesion, immunomodulation, antibacterial activity, and lipid metabolism (Supplementary Table S1).

The genomic features of the 10 *L. plantarum* reference strains with detailed WGS data are presented in Table 1. Each strain contained a circular chromosome and a different number of plasmids. The genome size of the reference strains was 3.2–3.6 Mp, the number of plasmids varied from 0 to 10, and the number of CDSs ranged from 2,893 to 3,127. A total of 2,350 genes were identified in the core genome, and 1,157 genes were identified as unique genes (Figure 1C). Among the unique genes, *L. plantarum* Dad-13 had the most (346) of unique genes, whereas *L. plantarum* ATCC 14917 had the fewest (52) unique genes. Eighty-five unique genes were identified in the HOM2217 genome, with 34 functionally annotated. A selection of these genes associated with probiotic functions is listed in Supplementary Table S2. To understand the evolutionary relationships among *L. plantarum* strains, ML and ANI trees were constructed (Figures 1D,E). HOM2217 showed a close relationship with the strain *L. plantarum* 299v, but was located on a relatively separate branch. ANI > 95% indicated the same species (Jain et al., 2018). The ANI values indicated that the 10 strains belonged to the same species (>99%), and *L. plantarum* 299v was the closest neighbor to HOM2217 (99.93% of the ANI value).

**Table 1:** Comparison of the chromosomal properties of 10 *L. plantarum* strains.

Strain	HOM2217	299v	Dad-13	P-8	ATCC14917	UBLP-40	EM	Q180	HAC01	LMT1-48
Accession No.	CP152360.1	GCA_001888735.1	GCA_023547165.1	CP005942.2	GCA_000143745.1	GCA_003692725.1	CP037429.1	CP073753.1	CP029349.1	CP033888.1
Genome size (Mp)	3.3	3.3	3.4	3.2	3.2	3.3	3.6	3.2	3.2	3.3
No. of plasmids	1	unknown	unknown	7	unknown	unknown	10	0	1	4
G + C contents (%)	44.5	44.5	44.5	44.5	44.5	44.5	44	44.5	44.5	44.5
CDSs	3,104	3,241	3,325	2,893	2,983	3,127	3,019	2,935	3,005	3,097
tRNA	68	56	67	66	64	67	72	71	67	76
rRNA	16	3	16	15	2	8	16	16	16	16
ANI (%)	100	99.93	99.45	99.08	99.32	99.16	99.40	99.26	99.24	99.17

### 3.2 Survival under simulated gastrointestinal tract conditions

**Figure 2:**
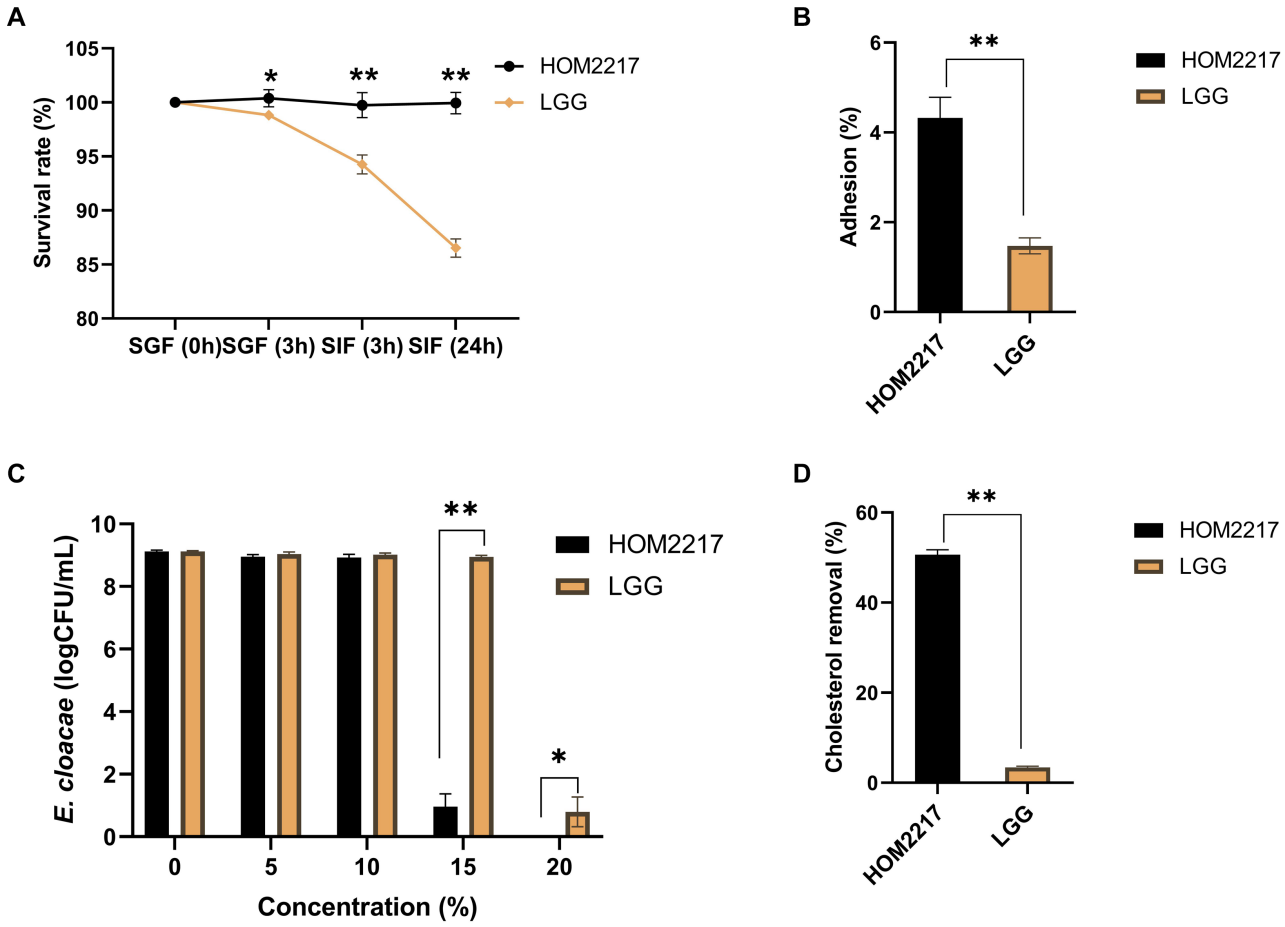
Characteristics of the HOM2217 strain. **(A)** The survival rate of HOM2217 under simulated gastrointestinal tract (GIT) conditions. SGF, simulated gastric fluid; SIF, simulated intestinal fluid. **(B)** Adhesion of HOM2217 to Caco-2 cells. **(C)** Antimicrobial activity of HOM2217 against the obesity-inducing bacterium *Enterobacter cloacae*. **(D)** Cholesterol removal (%) by HOM2217. *L. rhamnosus* GG was used as the control strain, error bars represent the mean ± SD (*n* = 3). *p < 0.05 and **p < 0.01.

The initial viable cell density in the simulated gastric acid was approximately 1 × 10^8^ CFU/mL. As shown in Figure 2A, HOM2217 had a significantly (*p* < 0.05) higher survival (100.39 ± 0.78%) in the simulated gastric fluid with pH of 3.0 for 3 h compared with that of *L. rhamnosus* GG (98.82 ± 0.46%). The final survival rates of HOM2217 following exposure to simulated intestinal fluid for 3 h and 24 h were 99.74 ± 1.15% and 99.95 ± 0.99%, respectively, which were significantly (*p* < 0.01) higher than those of *L. rhamnosus* GG.

### 3.3 Adhesion ability to Caco-2 cells

The adhesion of HOM2217 cells was expressed as the percentage of adhering isolates relative to the number of isolates added, as depicted in Figure 2B. The adhesion percentage of HOM2217 to Caco-2 cells after 2 h of cocultivation was 4.32 ± 0.46% which was significantly higher (*p* < 0.01) than that of *L. rhamnosus* GG.

### 3.4 Antimicrobial activity

Gram-negative *Enterobacter cloacae* strains were recently identified in obese mice and are considered pathogenic bacteria in the gut, resulting in obesity (Fei and Zhao, 2013). Therefore, a coculture experiment was performed to assess the antimicrobial activity of HOM2217 on *E. cloacae* ATCC 13047. The results are shown in Figure 2C. The number of *E. cloacae* only decreased by 0.16 and 0.19 orders of magnitude in the presence of 5 and 10% cell-free supernatants from HOM2217, respectively. However, the number of *E. cloacae* significantly decreased by 8.15 orders of magnitude when the 15% cell-free supernatant of HOM2217 was added. The growth of *E. cloacae* ATCC 13047 was completely inhibited when the cell-free supernatants of HOM2217 concentration was increased to 20%. The antimicrobial activity of HOM2217 against *E. cloacae* ATCC 13047 was significantly stronger (*p* < 0.05) than that of *L. rhamnosus* GG.

### 3.5 Assimilation of cholesterol

Bile tolerance and the ability to assimilate cholesterol are considered primary characteristics for the selection of potential probiotic strains with cholesterol-lowering effects. HOM2217 manifested superior ability (50.63 ± 1.08%) (Figure 2D) in the removal of cholesterol from the medium, and it was significantly (*p* < 0.01) higher than that of *L. rhamnosus* GG (3.42 ± 0.23%).

### 3.6 Assessment of the inhibitory effect on 3T3-L1 adipocytes

**Figure 3:**
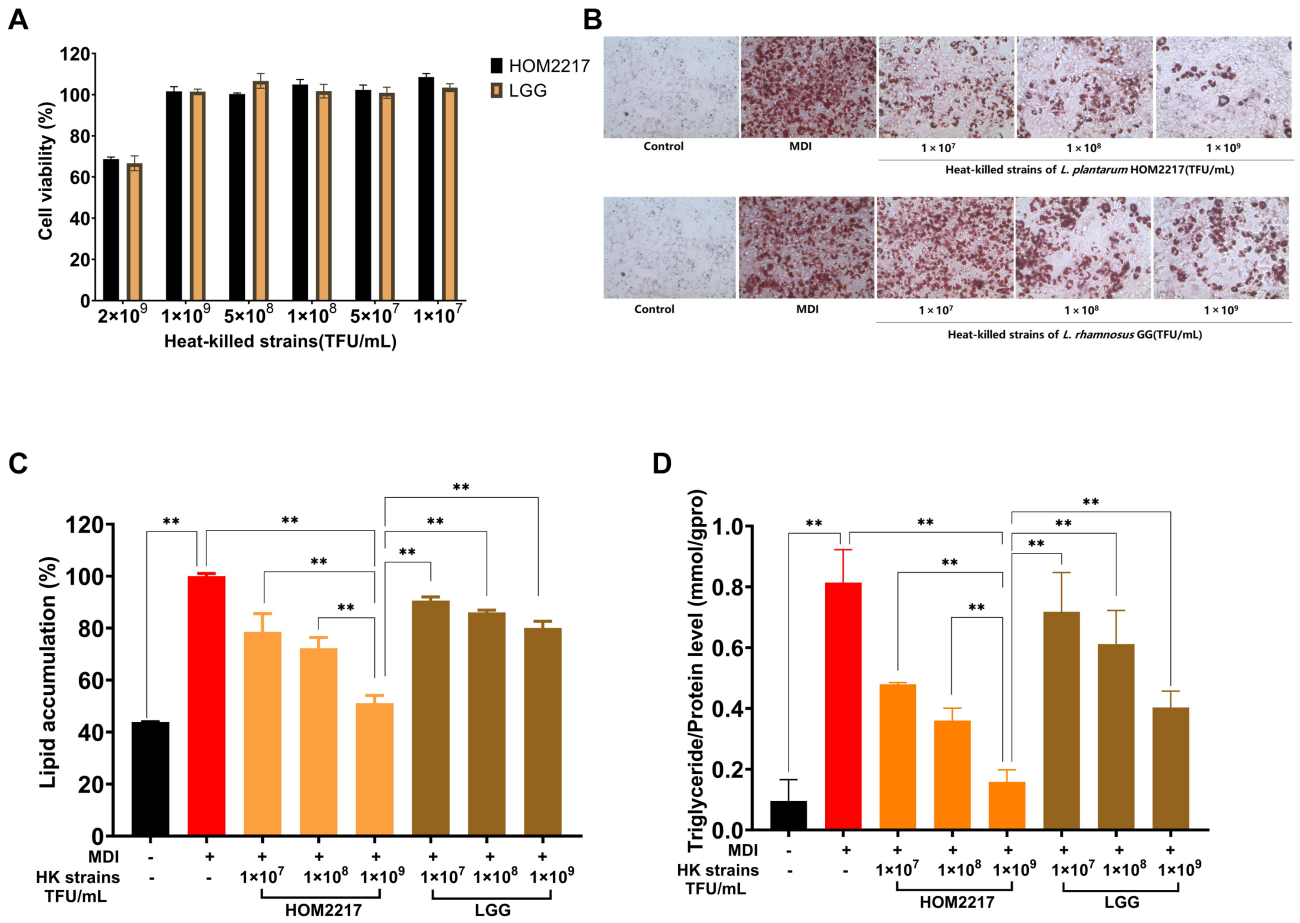
Effects of heat-killed strains of HOM2217 on lipid accumulation in 3T3-L1 adipocytes. **(A)** Cell viability, **(B)** Oil Red O staining, **(C)** Lipid accumulation, and **(D)** TG content. *L. rhamnosus* GG was used as the control strain. Error bars represent the mean ± SD (*n* = 3). *p < 0.05, **p < 0.01.

As shown in Figure 3A, the heat-killed strains exhibited no cytotoxicity against 3T3-L1 preadipocytes at concentrations of up to 1 × 10^9^ thallus forming units (TFU/mL). Thus, in subsequent experiments, heat-killed strain treatments were performed at ≤1 × 10^9^ TFU/mL. Heat-killed HOM2217 and *L. rhamnosus* GG were obtained by suspending the heat-killed cells in DPBS at 1 × 10^9^, 1 × 10^8^, and 1 × 10^7^ TFU/mL and then were used to treat 3T3-L1 preadipocytes during the adipogenic phase. 3T3-L1 adipocytes stained with Oil Red O solution were observed under an optical microscope (Figure 3B), which showed that more lipid droplets were formed in cells treated with MDI only, and lipid accumulation was also significantly (*p* < 0.01) increased. An eight-day treatment with heat-killed strains of HOM2217 or *L. rhamnosus* GG inhibited lipid accumulation reduction in a dose-dependent manner (21.4 ± 6.97% and 9.37 ± 1.41% at 1 × 10^7^ TFU/mL, 27.68 ± 4.10% and 13.93 ± 0.90% at 1 × 10^8^ TFU/mL and 48.80 ± 2.92% and 19.89 ± 2.53% at 1 × 10^9^ TFU/mL, respectively; Figure 3C). Based on the AdiopoRed assay, the addition of heat-killed strains of HOM2217 at 1 × 10^9^, significantly decreased lipid accumulation by 48.80 ± 2.92%, which was significantly (*p* < 0.01) better than the effect of other treatments (Figure 3C). The TG content of differentiated 3T3-L1 cells was the most repressed by 1 × 10^9^ TFU/mL of HOM2217 (Figure 3D). The degree of adipocyte differentiation inhibition was concentration-dependent on the heat-killed strain and was more pronounced with HOM2217 compared to *L. rhamnosus* GG, indicating that HOM2217 inhibits lipid accumulation in differentiated 3T3-L1 adipocytes.

### 3.7 Anti-obesity effect in diet-induced obese rats

**Figure 4:**
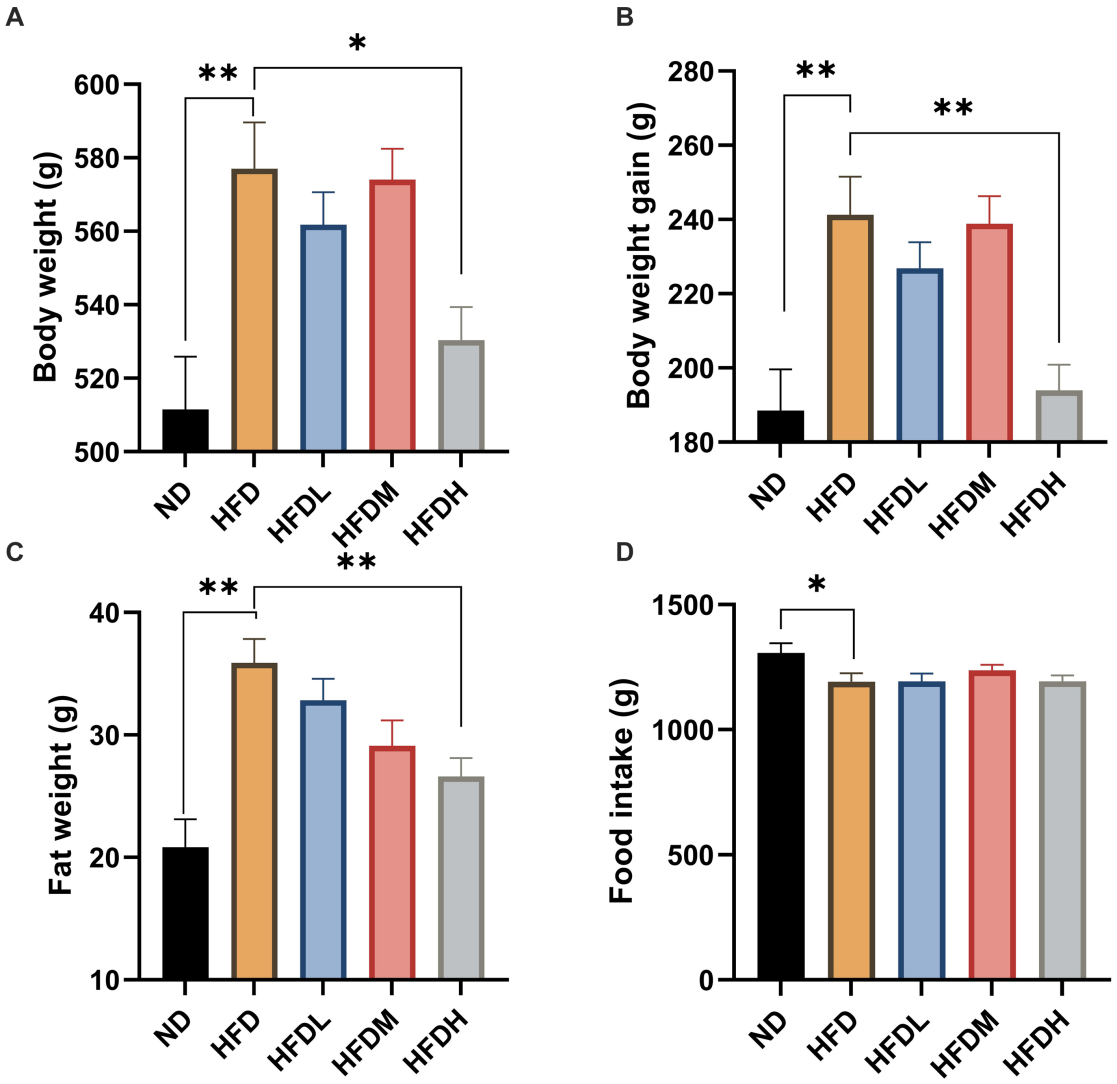
Anti-obesity effects of HOM2217 in high-fat diet-fed rats. **(A)** Body weight, **(B)** Body weight gain, **(C)** Fat weight, **(D)** Food intake. Error bars represent mean ± SEM (*n* = 10). *p < 0.05 and **p < 0.01. ND, normal diet; HFD, high-fat diet; HFDL, high-fat diet with low dose HOM2217 (2.5 × 10^9^ CFU/kg body weight); HFDM, high-fat diet with medium dose HOM2217 (1.25 × 10^10^ CFU/kg body weight); HFDH, high-fat diet with high dose HOM2217 (2.5 × 10^10^ CFU/kg body weight).

High-fat diet feeding induced significant (*p* < 0.01) body weight, body weight gain, and body fat throughout the study period compared to the ND (Figures 4A–C). The administration of high doses of HOM2217 induced lower body weight (*p* < 0.05), body weight gain (*p* < 0.01), and body fat (*p* < 0.05) compared to the HFD. However, the medium or low doses did not exhibit this effect. Interestingly, the reduction in body weight, body weight gain, and body fat in the HFDH group did not result from differences in food intake (Figure 4D).

**Figure 5:**
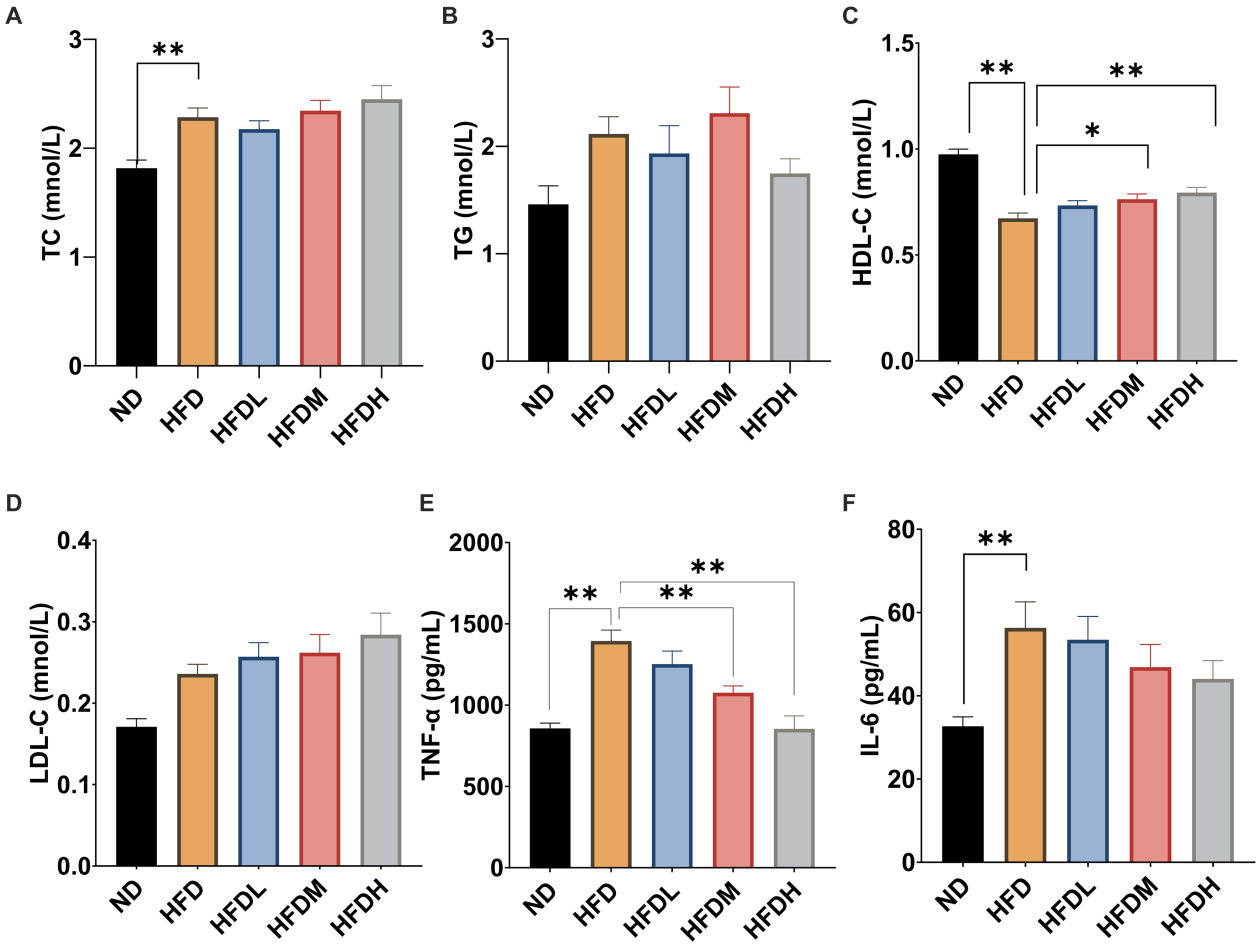
Effects of HOM2217 on blood lipid and cytokine levels in HFD-fed rats. **(A)** Serum total cholesterol (TC) level, **(B)** Serum triglyceride (TG) level, **(C)** Serum high-density lipoprotein cholesterol (HDL-C) level, **(D)** Serum low-density lipoprotein cholesterol (LDL-C) level, **(E)** Serum tumor necrosis factor (TNF-α) level, and **(F)** Serum interleukin 6 (IL-6) level. Error bars represent mean ± SEM (*n* = 10). *p < 0.05, **p < 0.01. ND, normal diet; HFD, high-fat diet; HFDL, high-fat diet with low dose HOM2217 (2.5 × 10^9^ CFU/kg body weight); HFDM, high-fat diet with medium dose HOM2217 (1.25 × 10^10^ CFU/kg body weight); HFDH, high-fat diet with high dose HOM2217 (2.5 × 10^10^ CFU/kg body weight).

As shown in Figure 5A, the serum total cholesterol concentrations were significantly higher in the HFC group than in the ND group (*p* < 0.01). However, there were no significant effects of HOM2217 supplementation on the TC concentration (*p* > 0.05). Serum TG levels were reduced by 17% in the HFDH group compared to those in the HFD group, but the difference was not significant owing to a large error (Figure 5B). Serum HDL-C concentrations were significantly increased both in the HFDM (*p* < 0.05) and HFDH groups (*p* < 0.01) compared to those in the HFD group (Figure 5C). There were no significant effects of HOM2217 supplementation on the LDL-C concentration (*p* < 0.05) (Figure 5D). As seen in Figures 5E,F, the serum cytokine indicators TNF-α and IL-6 were markedly higher in the HFD group than in the ND group (*p* < 0.01). Both the HFDM (*p* < 0.01) and HFDH groups (*p* < 0.01) had a significantly decreased serum TNF-α level but not serum IL-6 level.

**Figure 6:**
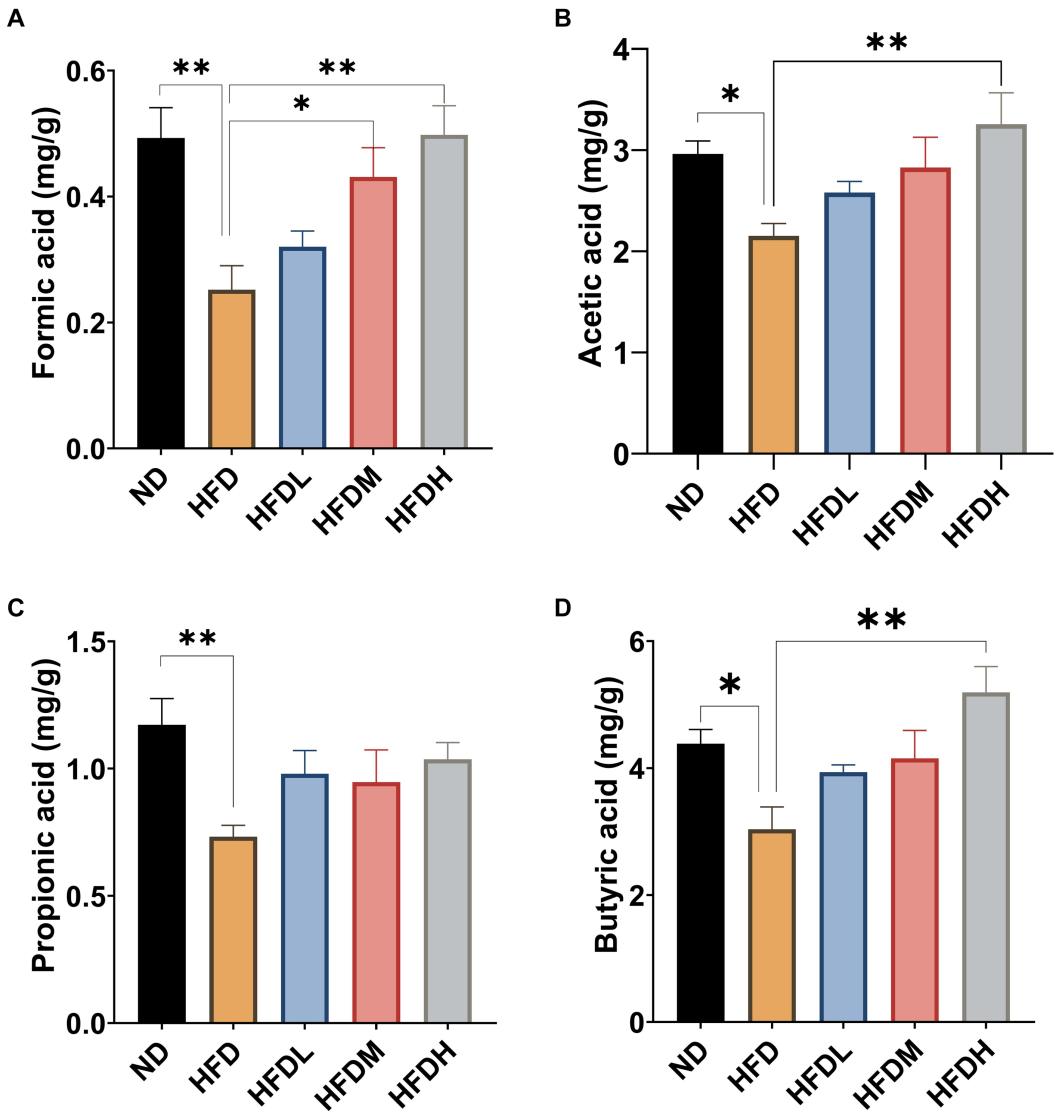
Effects of HOM2217 on cecum SCFAs concentrations in HFD-fed rats. **(A)** Formic acid, **(B)** Acetic acid, **(C)** Propionic acid, and **(D)** Butyric acid. Error bars represent mean ± SEM (*n* = 10). *p < 0.05, **p < 0.01. ND, normal diet; HFD, high-fat diet; HFDL, high-fat diet with low dose HOM2217 (2.5 × 10^9^ CFU/kg body weight); HFDM, high-fat diet with medium dose HOM2217 (1.25 × 10^10^ CFU/kg body weight); HFDH, high-fat diet with *high dose* HOM2217 (2.5 × 10^10^ CFU/kg body weight).

As shown in Figure 6, the concentrations of formic acid (*p* < 0.01), acetic acid (*p* < 0.05), propionic acid (*p* < 0.01), and butyric acid (*p* < 0.05) were significantly lower in the HFD group than in ND group. Supplementation with a high dose of HOM2217 significantly (*p* < 0.01) increased the concentrations of formic acid, acetic acid, and butyric acid, but did not significantly change the concentrations of propionic acid. The medium concentration of HOM2217 also significantly increased formic acid concentrations compared with the HFD group (*p* < 0.05) (Figure 6A).

## 4 Discussion

Obesity poses a major public health problem in all age groups worldwide; therefore, there is an urgent need to implement obesity prevention programs and identify new therapeutic and intervention targets. Previous study has demonstrated that several probiotics, used alone or in symbiotic mixtures, exert anti-obesity effects through species-and strain-specific mechanisms. In particular, *L. rhamnosus* GG supplementation 1 month before the expected delivery and subsequent treatment of the baby for a further 6 months lowered weight gain (age range 1–4 years) (Luoto et al., 2010). *L. rhamnosus* GG warrants consideration as a therapeutic tool to treat hypertransaminasemia in children with hepatopathic obesity who are noncompliant with lifestyle interventions (Vajro et al., 2011). *L. rhamnosus* GG inhibits fat accumulation in the liver by phosphorylating of adenosine monophosphate kinase (AMPK) (Zhang et al., 2015). A randomized, double-blind, placebo-controlled trial showed that the administration of *L. plantarum* LMT1-48 decreased body weight, abdominal visceral fat area, insulin resistance, and leptin levels in overweight subjects by regulating the gut microbiota (Sohn et al., 2023). In addition, *L. plantarum* Dad-13 significantly decreased body weight and BMI (*p* < 0.05) by decreasing the abundance of *Firmicutes* and increasing that of *Bacteroidetes* (particularly *Prevotella*) (Rahayu et al., 2021).

In this study, we isolated a novel strain from human milk, HOM2217, and analyzed its whole-genome sequence. The results showed that HOM2217 cells were identified as *L. plantarum. L. plantarum* is generally recognized as safe based on the long history of human consumption of *Lactobacilli* in food. It has been generally recognized as safe (GRAS) by the US Food and Drug Administration (FDA) and is included in the list of qualified presumptions of safety (QPS), recommended by the European Food Safety Authority (EFSA). To verify the probiotic characteristics of *L. plantarum* HOM2217 at the genomic level, genes related to probiotic properties were annotated using COG database. Based on published literature data, they reveal probiotic marker genes (PMG) which related to their characteristics (Chintakovid et al., 2024; Isaac et al., 2024). The proposed genes may represent strain-specific traits enhancing probiotic potential. The HOM2217 strain carries various probiotic marker genes, including four bsh genes (bsh1, bsh2, bsh3, and bsh4). Notably, bsh1 is primarily responsible for Bsh activity against pancreatin and low gastric pH and plays a role in lowering serum cholesterol levels (Lambert et al., 2008). This finding indicated its potential anti-obesity functions at genomic levels. Comparison with other *L. plantarum* strains revealed unique genes in HOM2217. For instance, the katE gene and its product, catalase, are crucial for the bacterium’s resistance to oxidative damage and its survival across various environmental conditions, including during infections and in the presence of host immune responses (Sun et al., 2022). In addition, our ANI tree results revealed that *L. plantarum* 299v and HOM2217 shared the highest similarity. As described, *L. plantarum* 299v is one of the most documented *L. plantarum* strains in the world, described in over 170 scientific publications, of which more than 60 are human clinical studies. It is safe, does not confer antibiotic resistance, and has been shown to provide relief of symptoms for those suffering from IBS and IBD and to mitigate iron deficiency (Nordstrom et al., 2021). In addition, Bouaziz et al. (2021) reported that the administration of *L. plantarum* 299v was useful in the treatment of obesity and metabolic syndrome in rabbits.

To achieve an anti-obesity effect in the intestine, probiotic bacteria should have the ability to pass through the upper parts of the gastrointestinal tract and adhere to intestinal epithelial cells, which helps them resist fluctuations in their intestinal levels and inhibit the attachment of pathogenic bacteria via competitive adhesion throughout the intestine. In this study, HOM2217 showed high tolerance to *in vitro* gastrointestinal conditions and exhibited high adhesive abilities to intestinal epithelial cell lines. *Enterobacteriaceae*, which are present in higher levels in obese subjects compared to non-obese subjects (Karlsson et al., 2012), can be used as an *in vitro* simulator of obesity. In this study, HOM2217 showed antimicrobial properties against *E. cloacae* ATCC 13047, indicating that HOM2217 may have a strong anti-obesity effect. Albano (Albano et al., 2018) tested the cholesterol removal capacity of seven *Lactobacillus casei* strains, 27 *Lactobacillus paracasei ssp. paracasei* strains, five *Lactococcus lactis* ssp*. lactis* strains, 15 *L. plantarum* strains, one *Enterococcus lactis* strain, and one *Enterococcus faecium* strain and reported that the lowest reduction in cholesterol was induced by *Lactococcus lactis* ssp. *Lactis*, whereas *L. plantarum* (26.1 ± 14.2%) and *Lactobacillus paracasei* ssp. *paracasei* (26.0 ± 10.4%) induced the highest decrease. In this study, HOM2217 showed a high cholesterol removal ability (50.63 ± 1.08%). Obesity is related to the differentiation, expansion, and lipid accumulation of adipocytes, and the degree of obesity is closely related to the degree of differentiation of preadipocytes into adipocytes and the enlargement of adipocytes in adipose tissues. HOM2217 significantly decreased TG content and lipid accumulation in fully differentiated 3T3-L1 adipocytes, reflecting its anti-obesity potential. Several studies have also found that *L. plantarum* species had an anti-obesity effect in 3T3-L1 cells, demonstrating that the inhibition of adipogenesis and lipid accumulation exerted by functional bacterial strains in mouse preadipocyte cells were mediated by the suppression of key adipogenic transcription factors (Park et al., 2011; Park et al., 2014; Jeung et al., 2018).

Our *in vitro* results indicated that treatment with heat-killed HOM2217 reduced lipid accumulation in adipocytes, prompting us to explore the anti-obesity effects of live HOM2217 *in vivo*. The consumption of a HFD is a key obesity-inducing factor, and long-term ingestion of HFD causes an increase in body fat in mammals. Therefore, in the present study, we investigated the anti-obesity effects of HOM2217 at three different doses in HFD-induced obese rats. We found that only high dose of HOM2217 exhibited anti-obesity effects against HFD-induced obesity, as demonstrated by the decreased body weight, body weight gain, and body fat without changes in food intake, which was in line with previous reports showing that only high dose of *L. plantarum* SKO-001 decreased the body weight and body fat in mice (Choi et al., 2023). The HDL-C level was higher than that of the HFD control group after supplementation with high and medium doses of HOM2217, indicating that HOM2217 helps regulate lipid metabolism in rats by increasing HDL-C levels. Recently, attention to inflammation in the intestine has been increasing since it was revealed that HFD induces inflammation by altering the gut microbiome and increasing intestinal permeability and may be a causative factor in the onset of obesity (Kim et al., 2012). We found that HOM2217 decreased serum TNF-α levels, which was supported by previous reports that *L. plantarum* ATG-K2 reduced the expression levels of inflammatory cytokines such as TNF-α and NF-κB in the small intestine of high-fat and high-fructose diet-fed rats (Park et al., 2021). Studies have also demonstrated that probiotics that produce short-chain fatty acids (SCFA) exhibit anti-obesity properties by regulating lipid and glucose metabolism, reducing adipocyte size, lowering cholesterol, and regulating leptin levels (den Besten et al., 2013). HOM2217 increased SCFA levels (formic, acetic, and butyric acids) in the cecum, which is consistent with the findings of Lee et al. (2021), who also found that gut microbiome modulation by *L. plantarum* ATG-K2 to increase SCFAs may serve as potential mechanism to alleviate obesity (Lee et al., 2021). However, due to the limitations of SCFAs produced by *L. plantarum*, further studies are needed to identify the effects of HOM2217 on the gut microbiota.

In summary, we sequenced the complete genome of HOM2217 isolated from human milk, studied its physiological characteristics, and investigated its anti-obesity effects in 3T3-L1 cells and HFD-fed rats to determine its potential as a starter for functional food products. Based on the complete genome sequence, the strain was named HOM2217, and was closest to *L. plantarum* 299v. The HOM2217 strain demonstrated greater tolerance to *in vitro* gastrointestinal conditions, higher adhesion to intestinal epithelial cell lines, superior antimicrobial activity against *E. cloacae* ATCC 13047, and more effective cholesterol removal compared to *L. rhamnosus* GG. In the present study, we found that HOM2217 exhibited anti-obesity effects in 3T3-L1 adipocytes and HFD-fed obese rats, which were accompanied by the inhibition of the inflammatory response and regulation of lipid metabolism and SCFAs levels. Hence, our results provide valuable insights into the potential of *L. plantarum* HOM2217 for suppressing obesity *in vitro* and in rats fed a high-fat diet. Taken together, these findings suggest that HOM2217 may has beneficial effects in ameliorating obesity and offer novel therapeutic strategies for obesity.

## Data Availability

The datasets presented in this study can be found in online repositories. The names of the repository/repositories and accession number(s) can be found in the article/Supplementary material.
