# Supplementary material for: Complete genome sequence and anti-obesity potential of Lactiplantibacillus plantarum HOM2217 in 3T3-L1 cells and high-fat diet-fed rats
Source: Front Microbiol. 2024 Sep 11;15:1436378. doi: 10.3389/fmicb.2024.1436378 (PMC11422070; doi:10.3389/fmicb.2024.1436378)
Supplement: Supplementary file 1 [file Table_1.docx]

Table S1 List of putative genes present in *L. plantarum* HOM2217 that are responsible for probiotic characteristics.

| Probiotic characteristic | Responsible genes in *L. plantarum* HOM2217 | COG ID | COG Description |
| --- | --- | --- | --- |
| Acid tolerance | clpL (Chr-gene3033) | COG0542 | ATP-dependent Clp protease, ATP-binding subunit ClpA |
|  | gadB (Chr-gene2898) | COG0076 | Glutamate or tyrosine decarboxylase or a related PLP-dependent protein |
|  | atpA (Chr-gene2044) | COG0056 | FoF1-type ATP synthase, alpha subunit |
|  | atpC (Chr-gene2041) | COG0355 | FoF1-type ATP synthase, epsilon subunit |
|  | atpD (Chr-gene2042) | COG0055 | FoF1-type ATP synthase, beta subunit |
|  | atpG (Chr-gene2043) | COG0224 | FoF1-type ATP synthase, gamma subunit |
|  | atpH (Chr-gene2045) | COG0712 | FoF1-type ATP synthase, delta subunit |
|  | copA (Chr-gene2609) | COG2217 | Cation-transporting P-type ATPase |
|  | guaA (Chr-gene0738) | COG0518 | GMP synthase, glutamine amidotransferase domain |
|  | plsC (Chr-gene1695) | COG0204 | 1-acyl-sn-glycerol-3-phosphate acyltransferase |
|  | pyk (Chr-gene1572) | COG0469 | Pyruvate kinase |
|  | recA (Chr-gene1983) | COG0468 | RecA/RadA recombinase |
| Bile tolerance | clpC (Chr-gene0846) | COG0542 | ATP-dependent Clp protease, ATP-binding subunit ClpA |
|  | cfa (Chr-gene2708) | COG2230 | Cyclopropane fatty-acyl-phospholipid synthase and related methyltransferases |
|  | argS (Chr-gene1165) | COG0018 | Arginyl-tRNA synthetase |
|  | ppaC (Chr-gene1523) | COG1227 | Inorganic pyrophosphatase/exopolyphosphatase |
|  | pgk (Chr-gene0623) | COG0126 | 3-phosphoglycerate kinase |
|  | dps (Chr-gene2675) | COG0783 | DNA-binding ferritin-like protein (oxidative damage protectant) |
|  | glnA (Chr-gene1302) | COG0174 | Glutamine synthetase |
|  | nagB (Chr-gene0200) | COG0363 | 6-phosphogluconolactonase/Glucosamine-6-phosphate isomerase/deaminase |
|  | pepO (Chr-gene2923) | COG3590 | Predicted metalloendopeptidase |
|  | pyrG (Chr-gene0416) | COG0504 | CTP synthase (UTP-ammonia lyase) |
|  | rplD (Chr-gene0856) | COG0088 | Ribosomal protein L4 |
|  | rplE (Chr-gene0867) | COG0094 | Ribosomal protein L5 |
|  | rplF (Chr-gene0869) | COG0097 | Ribosomal protein L6P/L9E |
|  | rpsC (Chr-gene0861) | COG0092 | Ribosomal protein S3 |
|  | rpsE (Chr-gene0871) | COG0098 | Ribosomal protein S5 |
| Stress tolerance | luxS (Chr-gene0618) | COG1854 | S-ribosylhomocysteine lyase LuxS, autoinducer biosynthesis |
|  | msrB (Chr-gene1522) | COG0229 | Peptide methionine sulfoxide reductase MsrB |
| Adherence or inhibition of pathogen adhesion | atpC (Chr-gene2041) | COG0355 | FoF1-type ATP synthase, epsilon subunit |
|  | dnaK (Chr-gene1664) | COG0443 | Molecular chaperone DnaK (HSP70) |
|  | htpX (Chr-gene0429) | COG0501 | Zn-dependent protease with chaperone function |
|  | hslO (Chr-gene0464) | COG1281 | Redox-regulated molecular chaperone, HSP33 family |
| Immunomodulation | dltA (Chr-gene1660) | COG1020 | EntF, seryl-AMP synthase component of non-ribosomal peptide synthetase |
|  | dltD (Chr-gene1657) | COG3966 | Poly-D-alanine transfer protein DltD, involved in esterification of teichoic acids |
|  | dltB (Chr-gene1659) | COG1696 | D-alanyl-lipoteichoic acid acyltransferase DltB, MBOAT superfamily |
| Antibacterial activity | agrA (Chr-gene0361) | COG3279 | DNA-binding response regulator, LytR/AlgR family |
|  | agrC (Chr-gene0360) | COG2972 | Sensor histidine kinase |
|  | blpA (Chr-gene0366) | COG2274 | ABC-type bacteriocin/lantibiotic exporters, contain an N-terminal double-glycine peptidase domain |
|  | blpB (Chr-gene0367) | COG1566 | Multidrug resistance efflux pump EmrA |
| Lipid metabolism | tagE (Chr-gene1100) | COG0438 | Glycosyltransferase involved in cell wall bisynthesis |
|  | rgpI (Chr-gene1703); gtrB (Chr-gene1200) | COG0463 | Glycosyltransferase involved in cell wall bisynthesis |
|  | bsh1 (Chr-gene2988); bsh2 (Chr-gene0067); bsh3 (Chr-gene2864); bsh4 (Chr-gene2210) | COG3049 | Penicillin V acylase or related amidase, Ntn superfamily |
|  | glpQ (Chr-gene2026) | COG0584 | Glycerophosphoryl diester phosphodiesterase |
|  | accB (Chr-gene0323) | COG0511 | Biotin carboxyl carrier protein |
|  | phnE (Chr-gene0562) | COG3639 | ABC-type phosphate/phosphonate transport system, permease component |
|  | adhE (Chr-gene0293) | COG1012 | Acyl-CoA reductase or other NAD-dependent aldehyde dehydrogenase |
|  | fabG (Chr-gene1383) | COG1028 | NAD(P)-dependent dehydrogenase, short-chain alcohol dehydrogenase family |

Table S2 The unique genes specific to *L. plantarum* HOM2217 through pan-genome comparison.

| Probiotic characteristic | Responsible genes in *L. plantarum* HOM2217 | COG ID | COG Description |
| --- | --- | --- | --- |
| Adherence | agrC (Chr-gene2615) | COG3290 | Sensor histidine kinase DipB regulating citrate/malate metabolism |
| Antioxidant | katE (Chr-gene0753) | COG0753 | Catalase |
| Chromate reduction | chrR (Chr-gene2768) | COG0431 | NAD(P)H-dependent FMN reductase |
| Antibacterial activity | PurL (Chr-gene2327) | COG0046 | Phosphoribosylformylglycinamidine (FGAM) synthase, synthetase domain |
| Carbohydrate uptake and catabolism | lacI (Chr-gene2949) | COG1609 | DNA-binding transcriptional regulator, LacI/PurR family |
| Converting L-rhamnose to L-rhamnulose | rhaA (Chr-gene2949) | COG4806 | L-rhamnose isomerase |
